# Post-isometric exercise hypotension occurs irrespective of muscle mass in adults with hypertension: A randomized clinical trial

**DOI:** 10.1016/j.clinsp.2025.100612

**Published:** 2025-04-16

**Authors:** Patrícia Caetano de Oliveira, Thiago Dipp, Gustavo Waclawovsky, Alexandre Machado Lehnen

**Affiliations:** a Instituto de Cardiologia do Rio Grande do Sul/Fundação Universitária de Cardiologia, Porto Alegre, RS, Brazil; b Programa de Pós-Graduação em Saúde Coletiva, Universidade do Vale do Rio dos Sinos, São Leopoldo, RS, Brazil

**Keywords:** Resistance exercise, Physical exercise, Upper limb, Low limb, Blood pressure, Ambulatory blood pressure monitoring

## Abstract

**Objective:**

Isometric exercise can be an alternative approach to lowering blood pressure in individuals with hypertension. However, it is uncertain whether the muscle mass involved can influence Post-Exercise Hypotension (PEH). Thus, the authors evaluate the acute effect of an isometric exercise session with a Small Muscle Mass (SMM) and a Large Muscle Mass (LMM) on Systolic (SBP) and Diastolic (DBP) blood pressure in individuals with hypertension.

**Method:**

Randomized clinical trial with 36 volunteers aged 40‒70y with hypertension allocated into (*n* = 12/group): control group (no exercise), SMM group (bilateral isometric handgrip contractions using a hydraulic handheld dynamometer), and LMM group (bilateral isometric full knee extension on a leg extension machine). Both exercise interventions consisted of 4 × 2 min of bilateral contraction at 30 % of the individual's maximum capacity. The authors used 24-hour Ambulatory Blood Pressure Monitoring (ABPM) after the interventions. One-way ANOVA or Generalized Estimation Equations (GEE) were used to test for potential differences (*p* < 0.05).

**Results:**

The authors found no increase in SBP/DBP immediately after the interventions. However, 24 h ABPM revealed lower SBP post-intervention in the SMM group (Δ-4.1 mmHg; *p* = 0.044) and the LMM group (Δ-5.6 mmHg; *p* = 0.040) compared to the control group, with no difference between these groups. DBP did not change over a period of 24 h ABPM.

**Conclusions:**

In conclusion, isometric exercise-induced PEH assessed by 24 h ABPM regardless of the muscle mass involved. This study shows that both interventions may contribute to lowering blood pressure in individuals with hypertension. Trial registration: www.clinicaltrials.gov; ID NCT03982758.

## Highlights

• SBP was lower in the SMM (−4.1 mmHg) and the LMM (−5.6 mmHg) vs. control.
• No statistical difference between SMM and LMM was observed.
• DBP did not change over a period of 24 h in response to SMM or LMM involved.
• Isometric exercise caused lower SBP, but not DBP, regardless of the mass of muscle.

## Introduction

Blood Pressure (BP) levels are directly associated with adverse cardiovascular events, i.e., high BP is linked to cardiovascular risk.^1^ BP levels should be reduced a normal range, and this requires lifestyle changes. Regular aerobic exercise is a well-established first-line nonpharmacological intervention for managing hypertension.^2^^,^^3^ Still, dynamic resistance exercise is also recommended as an adjunct to aerobic exercise and should be encouraged,^3^, ^4^, ^5^ and the recommendations regarding handgrip remain uncertain. Among many clinical practice guidelines for the management of HTN (Brazil, Canada, USA, and European Society of Cardiology), only the American College of Cardiology/American Heart Association Task Force on Clinical Practice Guidelines included isometric resistance exercise as a nonpharmacological intervention for the prevention and management of HTN.^3^ Still, the Brazilian Guidelines of Hypertension (2020) and the European Society of Cardiology (ESC) Council on Hypertension (2018 and 2023) just pointed out that both dynamic and isometric resistance training are “alternative exercise modalities” and can be recommended for secondary prevention of cardiovascular events in individuals with hypertension.^1^^,^^6^^,^^7^

The lack of consensus is based on the uncertainty regarding the effectiveness of isometric exercise in promoting Post-Exercise Hypotension (PEH) both as an acute effect and a chronic effect. Some studies have reported positive BP response following a single session of isometric exercise^8^, ^9^, ^10^ while others did not find this same benefit.^11^, ^12^, ^13^, ^14^ One aspect that may play a major role in BP response is the muscle mass involved in isometric exercise.

Evidence shows a reduction in SBP following dynamic whole-body resistance exercise.^15^^,^^16^ Polito et al.^17^ observed a reduction in SBP with PEH occurring only when exercise involved large muscle mass (leg extension). MacDonald et al.^18^ reported a direct effect of the muscle mass on the duration of response with prolonged PEH following leg exercise. Silvester^19^ assessed the effect of large and small muscle mass isometric exercise on BP response and found no PEH following leg press and handgrip isometric exercise. One hypothesis for the influence of muscle mass is its vasodilation capacity, which impacts blood pressure, though there is no consensus in the literature.^20^^,^^21^ Thus, studies that compared SBP/DBP response following exercise involving muscle mass of different sizes apparently did not find the superiority of large compared to smaller muscle groups. However, this evidence was gathered from samples of volunteers with normal BP^15^^,^^17^^,^^19^ or pre-hypertension^18^ and only one study examined isometric exercise (in volunteers with normal BP).^19^

Thus, to the best of our knowledge, most studies evaluated isometric exercise using a handgrip (a small muscle mass), and few studies have evaluated isometric exercise of larger muscle groups such as the quadriceps. The present study aimed to evaluate the effect on BP levels of a single bout of isometric exercise either involving a small muscle mass (using a handgrip) or a large muscle mass (on a leg extension machine) in individuals with hypertension. The authors hypothesized that both exercise interventions will induce PEH over a period of 24 h compared to a control session and that a single LMM session will generate PEH of greater magnitude compared to the SMM session.

## Materials and methods

The authors conducted a randomized, evaluator-blinded, parallel-group, exploratory clinical trial to evaluate the influence of muscle mass on BP response following a single bout of isometric exercise. The primary outcomes were SBP and DBP levels and the secondary outcomes were stroke volume, cardiac output, Pulse Wave Velocity (PWV) (arterial stiffness), and Peripheral Vascular Resistance (PVR).

The study was carried out at the Clinical Research Laboratory of Instituto de Cardiologia do Rio Grande do Sul/Fundação Universitária de Cardiologia (ICFUC). This research study follows the guidelines of the CONSORT Statement and the Declaration of Helsinki. It was approved by ICFUC Research Ethics Committee (# UP5515_18, October 2nd, 2018) and registered on Clinical Trials.gov (www.clinicaltrials.gov; ID NCT03982758). All participants read and signed an informed consent form before entering the study.

### Study participants

**Fig. 1:**
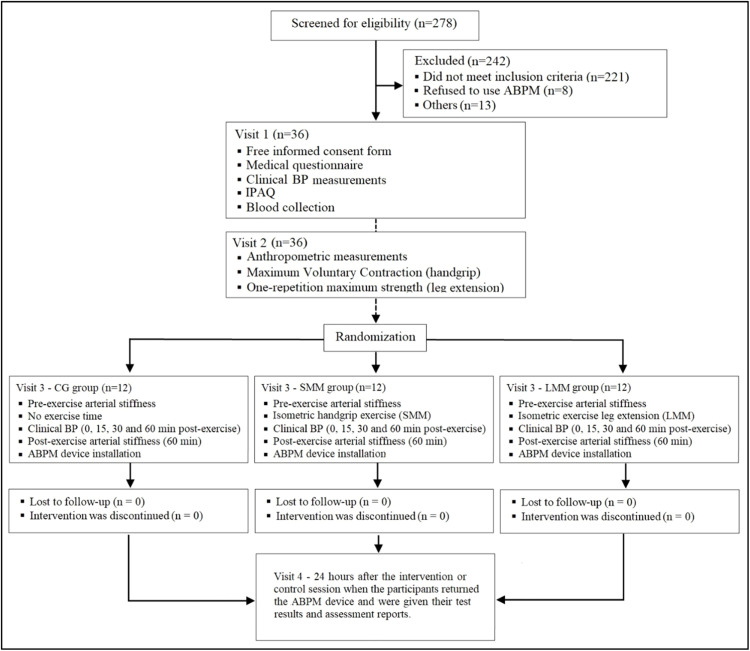
**Study design.** ABPM, 24-hour ambulatory blood pressure monitoring. BP, Blood Pressure; IPAQ, International Questionnaire of Physical Activity; CG, Control Group; SMM group, Small Muscle Mass (isometric handgrip exercise); LMM group, Large Muscle Mass (isometric knee extension exercise).

The study was conducted between August 2019 and February 2020. Recruitment was carried out through the ICFUC patient database as well as public notices on social media, totaling 278 eligible individuals – Fig. 1.

Low levels of physical activity were a criterion for inclusion in the study, i.e., < 600 MET-min/week as defined in the International Physical Activity Questionnaire (IPAq) (http://www.ipaq.ki.se/). There were excluded pregnant women and individuals with unstable angina, heart failure, recent cardiovascular events (in the last 3-months), chronic renal failure, history of a condition with a life expectancy < 2-years or any limitations that would prevent exercise, as well as those with resistant hypertension – uncontrolled resting BP despite the use of three or more antihypertensive drugs of different classes, including a diuretic, or the use of four or more drugs. All participants were evaluated for hypertension by a cardiologist at the studied site. Hypertension was confirmed when SBP ≥ 40 mmHg or DBP ≥ 90 mmHg according to the VII Brazilian Guidelines for Hypertension, and/or they reported the use of at least one of the following medications: angiotensin II receptor blockers; Angiotensin-Converting Enzyme (ACE) inhibitors; diuretics; betablockers; antiplatelet drugs and/or calcium channel blockers.

The authors estimated the sample size based on a reduction in SBP of 11.0 ± 5.8 mmHg,^22^^,^^23^ β = 90 % and α = 0.05. Thus, the final sample size was 36 individuals with *n* = 12 per group. To support the sample size calculation, the calculated Cohen's D effect size with a moderate effect was 0.7, with three groups and three repeated measures, β = 90 % and α = 0.05, also totaling 36 individuals.

### Randomization and groups

Randomization of the study interventions (exercise groups) was performed with the use of a computer program (www.randomization.org) with a coded numeric distribution (1‒2‒3). Allocation concealment was guaranteed; the random allocation of participants was kept in an inaccessible place and researchers did not have a priori knowledge of the intervention assignment to each participant. A numeric sequence was generated by a researcher blinded to the study for the individuals meeting the inclusion criteria. The numeric sequence for randomization was kept confidential until the very beginning of the experiment. Thus, thirty-six participants were randomized to three session groups: 1) Control session group or 2) Isometric handgrip exercise group (small muscle mass, SMM) or 3) Isometric knee extension exercise group (Large Muscle Mass, LMM) – Fig. 1.

### Study visits

The study consisted of four visits. On visit 1 the participants were asked to sign a free informed consent form, the IPAq was administered, and blood was drawn for 12 h fasting blood tests.

On visit 2 anthropometric measurements were taken (total body weight, height and waist circumference measurements) (see “Anthropometric measurements” section), and the participants underwent an assessment of Maximum Voluntary Contraction (MVC) using a handgrip or one-Repetition Maximum strength (1-RM) test on a leg extension machine (see “Maximum voluntary contraction and one-Repetition Maximum [1-RM] test” section).

On visit 3 the participants’ resting BP was measured and they underwent an assessment of pre-exercise arterial stiffness (see “Arterial stiffness” section). They were then asked to perform the exercise session (see “Intervention protocol” section) or a control session. BP levels were measured immediately after exercise (2‒3 min) and at 15-, 30- and 60-minutes post-exercise (see “Clinical assessment of BP” section). Post-exercise arterial stiffness was assessed immediately after BP recordings at 60 min. Then an Ambulatory Blood Pressure Monitoring (ABPM) device was placed on the participants (see “Ambulatory BP” section).

Visit 4 was scheduled for 24 h after the intervention or control session when the participants returned the ABPM device and were given their test results and assessment reports.

All visits followed a similar schedule: visit 1 took place from 8‒10 a.m. requiring fasting for blood collection; visit 2 was held 24‒48 h after visit 1 from 8‒10 a.m.; visit 3 was held 48‒72 h after visit 2 at 2 p.m. sharp; and visit 4 followed a flexible schedule.

The participants were instructed to eat light meals and avoid any vigorous physical activity, caffeinated and alcoholic beverages for 24 h before visit 2 (MVC and 1-RM test) and visit 3 (intervention/control session). Importantly, they maintained their routine use of antihypertensive drugs as prescribed.

### Anthropometric measurements

Total body weight and height of the participants were measured while wearing light clothing and no shoes to calculate Body Mass Index (BMI). The authors used a digital scale with a stadiometer attached (Welmy W200, São Paulo, Brazil). Waist circumference was measured by using a measuring tape at the smallest point between the rib and the iliac crest (Sanny, São Paulo, Brazil).

### Maximum voluntary contraction and one-Repetition maximum (1-RM) test

MVC was measured using a hydraulic hand dynamometer (Jamar, Chicago, USA) following the American Society of Hand Therapists recommendations. Briefly, the participants were asked to sit in a chair with a backrest, feet flat on the floor, position their shoulders in neutral rotation with the elbow flexed at 90°, the forearm in half pronation and the wrist in neutral, and hold the dynamometer in one hand with the arm away from their body. The handgrip of each hand was then measured using the hand dynamometer. They were instructed to perform a maximal isometric contraction for 5 s on the hand dynamometer, and each participant performed three attempts with a one-minute interval between attempts, and the highest value was recorded as MVC.

Muscle strength of lower limbs was tested bilaterally using a one-Repetition Maximum test (1-RM) based on previous studies of the research group.^24^^,^^25^ Briefly, the participants saw a demonstration and then were asked to practice exercising the quadriceps with full knee extension (180°) on a leg extension machine (Movement, Porto Alegre, Brazil). Prior to the 1-RM test, the participants performed a general warm-up (low-intensity biking for 3‒5 min) and then a specific warm-up consisting of 8 to 12 repetitions at 20 %‒25 % of the estimated load of 1-RM test at a cadence of 2 s per concentric and eccentric phase determined by an electronic metronome. After resting (3‒5 min), the 1-RM test was started. The participants then completed one repetition with increasing loads until they were no longer able to complete a single repetition. For successive attempts, 3‒5 min of rest were allotted between each attempt. The load at which they were unable to complete two repetitions was determined as the maximum exercise load (1-RM).

BP was monitored during the MVC and 1-RM test, as well as during the intervention sessions (see “Intervention protocol” section). Exercise sessions were not carried out when pre-intervention SBP/DBP was higher than 160/105 mmHg. Furthermore, the authors discouraged the Valsalva maneuver during muscle contractions. The Borg Rating of Perceived Exertion (6‒20 scale) was used to assess the subjective perception of effort during exercise (1‒2 min) and immediately after the exercise session (1‒2 min).

### Arterial stiffness

Central and peripheral BP and PWV were measured 5 min before and 60 min after each intervention or control session at the study site using a validated oscillometric BP measurement device (Mobil-O-Graph 24 h PWA Monitor®, IEM GmbH, Stolberg, Germany).^26^ All parameters were measured with the participants seated on a chair with their back supported against the backrest and feet flat on the floor in a quiet air-conditioned room (temperatures of 22°‒24 °C). They were asked to remain seated for 10 min before taking any measurements. The cuff was placed around the participant's arm and three readings were taken at 3-minute intervals (automated measurements with no operator intervention). The instrument measured and calculated stroke volume, cardiac output, central Systolic BP (cSBP), central Diastolic BP (cDBP) and PWV. PWV estimates were derived from in-built ARCSolver algorithms.^27^

### Intervention protocol

The participants in the SMM group performed 4 sets of 2-minute bilateral isometric contractions using a hydraulic hand dynamometer (Jamar, Chicago, USA) at 30 % of MVC, as follows:4×[2−minuteleft−handcontraction+2−minuteright−handcontraction+one−minuterest]. The evaluator adjusted manually the peak-hold needle to 30 % of MVC and then stood in a position that allowed a clear view of the handgrip display.

Those in the LMM group performed 4 sets of 2-minute bilateral isometric contractions with full knee extension on a leg extension machine (Movement, Porto Alegre, Brazil) at 30 % of 1-RM, and one-minute rest was allotted between each set.

The control group followed the same procedure, except for the intervention when they remained seated for the duration of the session.

### Blood pressure

#### Clinical assessment of BP

Resting BP measurements were taken with the participants seated on a chair with their back supported against the backrest and feet flat on the floor in a quiet air-conditioned room (temperatures of 22°‒24 °C). They were asked to remain seated for 10 min and then measurements were taken in triplicate on each arm at 1-min intervals using a semi-automatic device (OMROM® 705CP, São Paulo, Brazil). The average of these three measurements on each arm was recorded. When they were greater than 5 mmHg (for SBP/DBP), an additional measurement was taken to replace the discrepant value. The authors subsequently used the arm with the higher mean readings for BP measurements in triplicate throughout the study.

#### Ambulatory BP

Blood pressure monitoring devices (Spacelab, Redmond, WA, USA) were used to obtain 24-hour BP recordings following standard procedures. During ABPM, BP was recorded every 15 min between 7:00 a.m. and 10:00 p.m. and every 30 min during nighttime (from 11:00 p.m. to 6:00 a.m.). The authors considered an average sleep time of eight hours based on the participant's reported daily routine in the initial interview. When there were no ABPM readings for a full hour, the average values for the hour immediately before and after were recorded.

The participants were asked to keep a detailed record of their daily activities such as sleep, work, leisure, food, among others, for verification of any sudden changes in BP. They were instructed to whenever possible adjust their normal routine to daytime and nighttime (awake and sleep) periods on the ABPM device; they all agreed to follow this recommendation. In addition, they were strongly advised to avoid any physical activity and caffeinated and alcoholic beverages during the 24 h ABPM period (post-exercise or control session). ABPM measures were assessed by an evaluator blinded to the study interventions.

Since both the control and intervention groups had similar characteristics, so the authors assessed the hypotension main effect by comparing SBP/DBP of the SMM and LMM groups versus the control group, as well as BP in the SMM group versus the LMM group. Additionally, the authors evaluated BP within each group. These same procedures were applied to both clinic BP and ABPM measurements. In particular, for ABPM measurements, the authors assessed PEH for SBP/DBP over a period of 24 h (average of all times) and during daytime and nighttime separately.

### Statistical analysis

The Shapiro-Wilk and Levene's tests were used to test the assumption of normality and homogeneity of variance, respectively. Data were presented as means ± Standard Deviation (SD) as well as 95 % Confidence Intervals (95 % CI). Potential differences at baseline were tested by one-way analysis of variance (ANOVA) followed by Bonferroni's post-hoc tests. A Generalized Estimation Equations (GEE) of two factors (group, time and group × time interaction) were performed as needed followed by Bonferroni's post-hoc tests. SPSS v23 was used for conducting all statistical analyses (*p* < 0.05).

## Results

Of eligible individuals, 8 were excluded as they refused to undergo 24-hour ABPM and another 13 withdrew due to commuting and personal issues. Thus, the study sample consisted of 36 participants (14 men and 22 women: CG, 6 men + 6 women; SMM, 5 men + 7 women; LMM, 4 men + 8 women), mean age of 58.6 ± 10.0 years and BMI 30.2 ± 4.8 kg/m^2^ completed the study. At study entry, anthropometric parameters, lipid profile and glycated hemoglobin levels were similar among the groups (Table 1). However, fasting blood glucose was lower in the LMM group compared to the control group (Δ16.0 mg/dL; *p* = 0.013). Resting SBP, resting DBP, MVC and 1-RM did not show any differences among the groups. Table 2 shows a list of medications used by the participants. Briefly, 16 volunteers were taking one antihypertensive drug, 13 were taking a two-drug regimen and seven a three-drug regimen.

**Table 1:** Characteristics of the groups studied.

	**CG (*n* = 12)**	**SMM (*n* = 12)**	**LMM (*n* = 12)**	**p-value**
	**Mean ± SD (95 % CI)**	**Mean ± SD (95 % CI)**	**Mean ± SD (95 % CI)**	
**Age (years)**	57 ± 14 (48; 65)	59 ± 9 (53; 65)	60 ± 8 (55; 65)	0.722
**Height (cm)**	169 ± 10 (163; 176)	163 ± 8 (158; 168)	164 ± 9 (158; 170)	0.221
**Body weight (kg)**	88.1 ± 20.5 (75.1; 101.1)	79.9 ± 14.3 (70.8; 89.0)	82.9 ± 23.7 (67.8; 98.0)	0.602
**BMI (kg/m^2^)**	30.4 ± 4.7 (27.4; 33.3)	30.0 ± 3.8 (27.6; 32.5)	30.2 ± 5.9 (26.5; 34.0)	0.984
**Waist circumference (cm)**	94 ± 13 (85; 102)	95 ± 12 (88; 103)	96 ± 15 (86; 105)	0.916
**Fasting blood glucose (mg/dL)**	112 ± 21 (99; 126)	99 ± 7 (95; 103)	96 ± 6^a^ (91; 100)	0.011
**Total cholesterol (mg/dL)**	205 ± 49 (173; 236)	192 ± 12 (184; 200)	207 ± 43 (180; 234)	0.588
**HDL (mg/dL)**	51 ± 9 (45; 57)	52 ± 9 (47; 58)	50 ± 10 (44; 56)	0.813
**LDL (mg/dL)**	92 ± 43 (65; 119)	79 ± 9 (73; 84)	82 ± 8 (77; 88)	0.426
**Triglycerides (mg/dL)**	217 ± 73 (171; 264)	202 ± 20 (189; 214)	244 ± 56 (208; 280)	0.172
**Resting SBP (mmHg)**	135.9 ± 5.4 (124.8; 138.2)	136.3 ± 19.0 (124.2; 148.3)	135.3 ± 18.0 (123.8; 146.7)	0.873
**Resting DBP (mmHg)**	86.9 ± 7.8 (78.1; 97.3)	83.8 ± 10.8 (77.0; 90.7)	86.8 ± 13.3 (78.4; 95.3)	0.751
**MVC (kgf)**	26.6 ± 5.8 (21.2; 31.9)	26.0 ± 3.4 (23.8; 28.2)	29.5 ± 4.0 (27.0; 32.0)	0.124
**1-RM (kg)**	21.4 ± 8.2 (16.3; 26.6)	15.8 ± 13.9 (6.9; 24.6)	19.6 ± 7.0 (15.1; 24.0)	0.385

CG, Control Group; SMM, Small Muscle Mass (isometric handgrip exercise); LMM, Large Muscle Mass (isometric knee extension exercise); BMI, Body Mass Index; HDL, High Density Lipoprotein; LDL, Low Density Lipoprotein; SBP, Systolic Blood Pressure; DBP, Diastolic Blood Pressure; MVC, Maximum Voluntary Contraction; kgf, Kilogram-force; 1-RM, one-Repetition Maximum test. Any differences were tested by one-way analysis of variance (ANOVA) followed by Bonferroni's post-hoc tests.

a *p* < 0.05 vs. control group.

**Table 2:** Antihypertensive medications used by the study participants.

	Total (*n* = 36)	CG (*n* = 12)	SMM (*n* = 12)	LMM (*n* = 12)
**Angiotensin-receptor blockers**	18	3	8	7
**ACE inhibitors**	7	2	2	3
**Statins**	9	2	3	4
**Diuretics**	11	2	7	2
**Betablockers**	8	1	4	3
**Antiplatelets**	1	0	1	0
**Calcium channel blockers**	9	2	5	2

CG, Control Group; SMM, Small Muscle Mass (isometric handgrip exercise); LMM, Large Muscle Mas (isometric knee extension exercise); ACE, Angiotensin-Converting Enzyme.

**Fig. 2:**
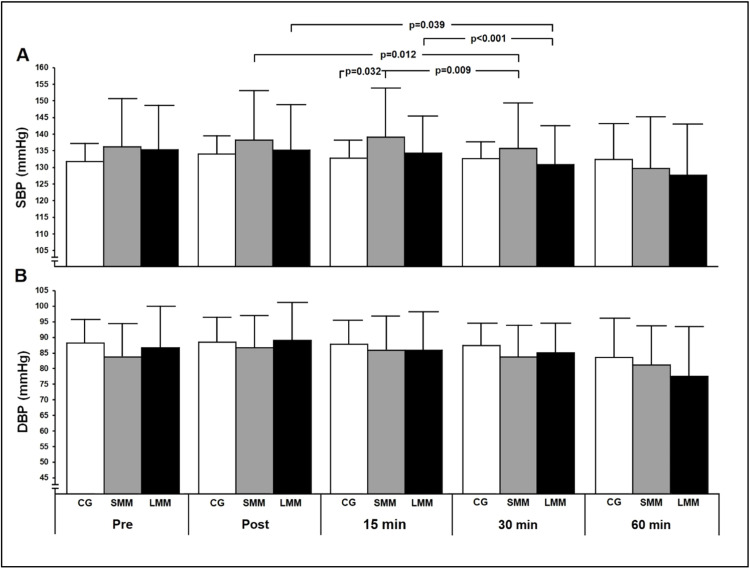
**Changes in blood pressure compared to the control group.** Panel A, Systolic Blood Pressure (SBP); Panel B, Diastolic Blood Pressure (DBP). Values are presented as mean ± SD. CG, control group (*n* = 12); SMM group (*n* = 12), small muscle mass (isometric handgrip exercise). LMM group (*n* = 12), large muscle mass (isometric knee extension exercise). Potential differences were tested by GEE (Generalized Estimation Equations) with two factors (group × time and group and time interaction). Bonferroni's post-hoc tests were performed when necessary. For SBP (Panel A), p(group) = 0.793; p(time) < 0.001; p(interaction) = 0.002. For DBP (Panel B): p(group) = 0.770; p(time) < 0.001; p(interaction) = 0.200.

Fig. 2 shows SBP and DBP values pre-intervention, immediately after the intervention, and at 15-, 30- and 60-minutes post-intervention. There was no increase in SBP and DBP levels at the end of the exercise session, but the SMM group showed higher SBP values at 15-minutes post-exercise when compared to the control group (*p* = 0.032). Also, after 30-minutes of exercise, these levels were similar to the control group. DBP values remained unchanged until 60-minutes post-exercise (*p* = 0.200) when compared to the control group.

No relevant hemodynamic changes were seen in stroke volume, cardiac output, PWV and PVR throughout the study (Table 3).

**Table 3:** Hemodynamic parameters and arterial stiffness.

	CG (*n* = 12)		SMM (*n* = 12)		LMM (*n* = 12)				
	Pre-exercise	60 min post-exercise	Pre-exercise	60 min post-exercise	Pre-exercise	60 min post-exercise	p-value (group)	p-value (time)	p-value (interaction)
**Stroke volume (mL)**	62.4 ± 11.3	67.3 ± 7.5	70.7 ± 14.0	71.2 ± 9.6	71.3 ± 17.4	74.5 ± 15.7	0.263	0.174	0.489
**Cardiac output (mL/min)**	5.4 ± 0.7	5.6 ± 0.5	5.3 ± 1.0	5.4 ± 0.9	5.4 ± 1.4	5.6 ± 0.6	0.738	0.898	0.480
**PWV (m/s)**	7.8 ± 1.3	7.9 ± 1.4	8.3 ± 1.1	8.4 ± 1.1	8.5 ± 1.0	8.6 ± 0.9	0.496	0.406	0.849
PVR (s*mmHg/mL)	1.14±0.19	1.14±0.06	1.17±0.18	1.20±0.13	1.10±0.17	1.15±0.18	0.436	0.389	0.839

CG, Control Group; SMM, Small Muscle Mass (isometric handgrip exercise); LMM, Large Muscle Mass (isometric knee extension exercise); PWV, Pulse Wave Velocity; PVR, Peripheral Vascular Resistance.

Values are presented as mean±SD. Potential differences were tested by GEE (Generalized Estimation Equations) with two factors (group × time and group and time interaction). Bonferroni's post-hoc tests were performed when necessary.

**Fig. 3:**
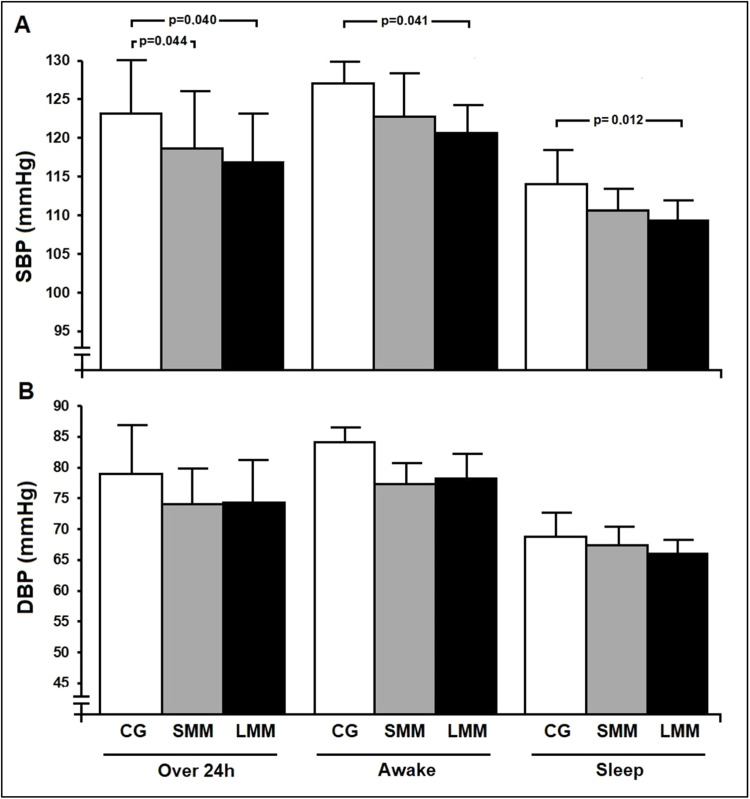
**Changes in blood pressure over 24-hour ABPM post-exercise**. Panel A, Systolic Blood Pressure (SBP); Panel B, Diastolic Blood Pressure (DBP). An entire 24-hour time period of Ambulatory Blood pressure Monitoring (ABPM) comprised readings during awake (7am to 10pm) and sleep (11am to 6am) periods. Values are presented as mean ± SD. CG, control group (*n* = 12); SMM group (*n* = 12), small muscle mass (isometric handgrip exercise); LMM group (*n* = 12), large muscle mass (isometric knee extension exercise). Potential differences among groups at the same time were tested by one-way analysis of variance (ANOVA) followed by Bonferroni's post-hoc tests; Panel A: over 24 h (*p* = 0.036), awake (*p* = 0.045) and sleep (*p* = 0.017) – *p* < 0.05 for all statistical tests.

**Fig. 4:**
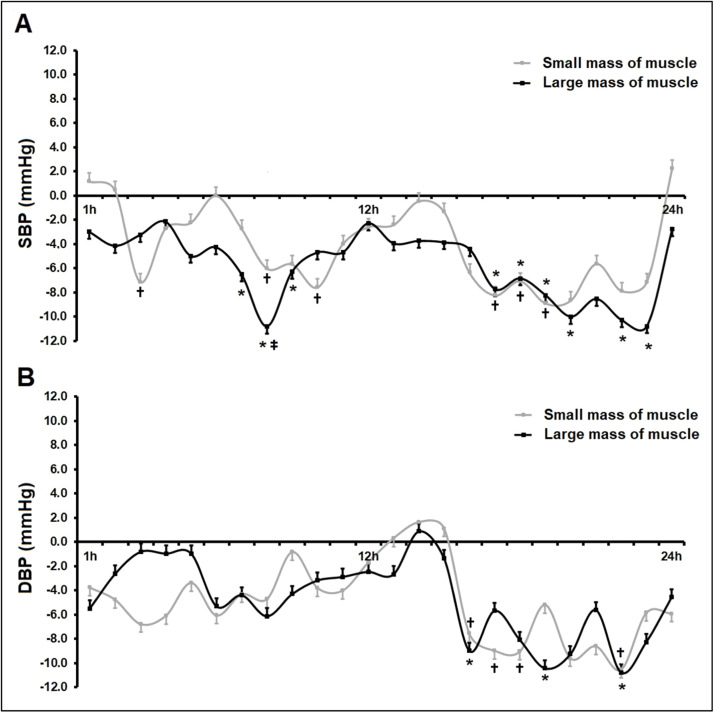
**Differences between the intervention groups (small or large muscle mass) compared to the control group over 24h Ambulatory Blood Pressure Monitoring (ABPM).** The chart shows changes in systolic blood pressure (SBP, Panel A) and Diastolic Blood Pressure (DBP, Panel B) in the SMM group (*n* = 12), small muscle mass (isometric handgrip exercise), and LMM group (*n* = 12), large muscle mass (isometric knee extension exercise) when compared to the control group, *n* = 12 (zero line). Values are presented as mean±SD. Potential differences were tested by GEE (generalized Estimation Equations) with two factors (group × time and group and time interaction). Bonferroni's post-hoc tests were performed when necessary. * and † *p* < 0.05 vs. control group (zero line); ‡ *p* < 0.05 vs. SMM group.

Fig. 3 shows mean SBP and DBP values over 24 h ABPM. All participants reported compliance with previously agreed time schedules for BP monitoring (awake and sleep periods). Systolic PEH occurred in the SMM group (Δ24hSBP −4.1 mmHg; *p* = 0.044) and the LMM group (Δ24SBP −5.6 mmHg; *p* = 0.040) when compared to the control group (Fig. 3, Panel A), with no differences between sessions (Δ24SBP −1.5 mmHg; *p* = 1.000). In addition, the authors did not see any relevant changes in DBP over 24 h of ABPM. When ambulatory BP values were analyzed on an hourly basis over 24 h post-exercise (Fig. 4), the authors found lower SBP values for both intervention groups compared to the control group (zero line) during most of the period assessed.

## Discussion

The main finding of the present study was that a single bout of isometric exercise reduced post-exercise SBP (PEH) as assessed by 24 h ABPM regardless of the muscle mass involved: −4.1 mmHg in the SMM group and −5.6 mmHg in the LMM group, with no differences between session. The findings of this study did not support that the amount of muscle mass involved in exercise influences hemodynamic responses.^28^^,^^29^ Thus, the authors accept the hypothesis that isometric exercise induces PEH over 24 h of the intervention, but the authors reject that exercise involving a larger muscle mass is more effective in lowering SBP/DBP.

Regarding clinical BP, the authors found no reduction in BP within 60 min post-exercise, which is consistent with that reported in other studies.^11^^,^^12^ This finding is in part supported by unchanged hemodynamic parameters (Table 3) as BP is defined as the product of cardiac output and peripheral vascular resistance. The most plausible explanation for this finding is the activation of the Sympathetic Nervous System (SNS) and endothelium-dependent vasodilation. During exercise, increased sympathetic activity induces arterial vasoconstriction.^30^ that is associated with a decrease in endothelium-dependent vasodilation post-exercise.^31^ There is an apparent transient biphasic response post-exercise.^32^ It is therefore possible that the SNS influences endothelium-dependent vasodilation up to 1-hour post-exercise.^32^ This assumption is supported by a trend of BP reduction over 60-minutes post-exercise. The authors can speculate that it could be statistically demonstrated over 24 h ABPM (from 60-minutes up to 24-hours).

As for BP variation over 24 h, a number of studies have assessed the effect of a bout of isometric handgrip exercise in individuals with hypertension.^11^, ^12^, ^13^^,^^33^^,^^34^ However, only two of them used ABPM^13^^,^^33^ to assess ambulatory BP values as in the present study. The authors found conflicting results when comparing this study to other studies that used ABPM. Ash et al.^13^ evaluated the acute effect of a session of aerobic exercise and isometric handgrip exercise versus a control session and they found no changes in BP post-isometric handgrip exercise. Van Assche et al.,^33^ on the other hand, reported a reduction in SBP. Although these findings are close to those reported by Van Assche, a direct comparison is difficult because of different sample profiles. The authors studied a sample of individuals with hypertension while these authors evaluated individuals with pre-hypertension. It is well-established that individuals with hypertension have higher baseline BP levels and evidence demonstrates greater reductions in SBP/DBP in populations with higher levels.^12^^,^^35^^,^^36^ The mechanism that may explain PEH over 24-hours in the present study is the association of endothelium-dependent vasodilation and BP levels. Both dynamic strength exercise^37^ and isometric exercise^38^ have been shown to increase endothelium-dependent vasodilation improving arterial compliance. It is thus possible that shear stress-induced Nitric Oxide (NO) production^32^ following exercise and/or blood vessel responsiveness to NO may have partially contributed to a similar pressure-lowering effect of isometric exercise in the SMM and LMM groups.

In regard to the muscle mass involved, the magnitude of adaptations to exercise stimulus may be related to the muscle mass tissue involved.^39^ Therefore, as the authors postulated, it is plausible to assume that the PEH effect would be greater in the LMM group than in the SMM group; however, these findings did not support this assumption. Millar et al.^40^ have also suggested that BP reductions following isometric exercise are not dependent on the muscle mass activated. Mechanisms involved in the regulation of BP, including exercise pressor reflex and endothelium-dependent vasodilation, may contribute to these effects in response to isometric handgrip exercise (SMM) and isometric exercise on a knee extensor machine (LMM) leading to PEH of similar magnitude. However, it is noteworthy that exercising at 30 % of 1-RM in the LMM group may have underestimated the intensity relative to 30 % of MVC with a lower amount of effort in the lower limb exercise session. Evidence showed lower intensity in lower limbs when compared to upper limbs,^41^ which is consistent with the methods used and the results found in this study. Thus, these findings suggest that a lower amount of isometric effort in the lower limbs, as in this study, could produce a similar response to handgrip isometric contractions in the upper limbs.

The present findings have clinical significance because a reduction in SBP, if maintained in the long run, can reduce the long-term risk of cardiovascular death.^3^^,^^5^ Some evidence exists to suggest that long-term reductions in SBP of >2‒5 mmHg are significantly important for the management of hypertension and may contribute to 14 % risk reduction of stroke mortality, 9 % of coronary disease, and 7 % of death from all causes.^40^^,^^42^ Moreover, the results of the present study are comparable to the expected PEH response to aerobic exercise (5‒7 mmHg) and are greater in magnitude than PEH induced by dynamic resistance exercise showing a mean reduction of 2‒3 mmHg.^4^^,^^43^ Still, there is no consensus in the literature regarding the expected magnitude of BP reduction in response to a bout of isometric exercise by handgrip or any other type of exercise. In addition, it is important to highlight that no adverse events (peak blood pressure during or after exercise sessions, muscle pain, discomfort, among others) were reported throughout the study, which further supports that this a safe, easy-to-perform exercise modality that does not require to spend much time.

Some limitations of this study should be noted. Although the authors estimated a sample size of 36 individuals as being sufficient to show potential BP differences (as mentioned in the “Study participants” section), the authors believe that a larger sample would allow us to stratify the groups by some variables, including age, sex, duration of hypertension and medication use, among others. Though the present study examines the acute effects of exercise, this sample was calculated based on a study of chronic effects. However, it should be noted there is a relationship between acute and chronic effects of exercise^44^^,^^45^ which can support the present choice for sample size calculation. In addition, the authors included pre- and post-menopause women who met the inclusion/exclusion criteria. Although pre- and post-menopause states may affect BP, the random assignment of participants reduces potential confounding effects. Another limitation is the use of 1-RM for isometric contraction in the LMM group because a large muscle mass is able to generate greater force than dynamic tension. Thus, the fact that LMM intensity was based on 1-RM test (dynamic strength) and not CVM (isometric strength) implies that although using 30 % for both exercises, the relative intensity may have been different between the exercise protocols. Also, while the participants in the SMM group alternated one-hand isometric contractions with a period of rest, those in the LMM group performed simultaneous knee extensions (as for 1-RM test) with a period of rest. This fact may imply the volume of effort: SMM was executed for 20-min [4 × (2 + 2 + 1)] and LMM for 12-min [4 × (2 + 1)]. However, these limitations do not invalidate these findings of PEH response and comparison of two different exercise modalities as per the study design, as well as the clinical relevance of the findings. On the contrary, the present study presents strengths that should be highlighted and minimize the limitations pointed out. The main strength is the use of 24-hour ABPM for BP assessments. The clinical relevance of PEH only makes sense as a cardioprotective effect when analyzed over at least 24-hours post-exercise. Short-term evaluations (15-, 30-, 60-minutes) are more associated with exercise safety than with cardioprotective effects (reduction in cardiac injury as myocardial infarction). Another strength of the present study is the homogeneity of the participants (Table 1), such as age, anthropometry (height, body weight, and BMI), as well as baseline blood pressure (SBP and DBP values). In this context, there was no concern about potential confounding effects on the primary outcome.

## Conclusion

The authors showed that one single bout of isometric exercise-induced PEH assessed by 24 h ABPM regardless of the muscle mass involved, with the cardiovascular load persisting reduced for many hours, imposing less stress and may be lower risk for the participants. Therefore, the practice of isometric exercise involving small or large muscle mass can be considered as an alternative to the treatment of hypertension.

## Funding

The authors received no financial support for the research, authorship, and/or publication of this article.

## Declaration of competing interest

The authors declare no conflicts of interest. They all have read and approved the final manuscript.

## Appendix Supplementary materials

Image, application 1
